# Serum Fatty Acid Reference Ranges: Percentiles from a New Zealand National Nutrition Survey

**DOI:** 10.3390/nu3010152

**Published:** 2011-01-20

**Authors:** Kathryn E. Bradbury, Clark Murray Skeaff, Francesca L. Crowe, Timothy J. Green, Leanne Hodson

**Affiliations:** 1 Department of Human Nutrition, University of Otago, Dunedin 9054, New Zealand; Email: braka554@student.otago.ac.nz; 2 Cancer Epidemiology Unit, Nuffield Department of Clinical Medicine, University of Oxford, Oxford, UK; Email: francesca.crowe@ceu.ox.ac.uk; 3 Faculty of Land and Food Systems, the University of British Columbia, Vancouver, British Columbia, Canada; Email: tigreen@interchange.ubc.ca; 4 Oxford Centre for Diabetes, Endocrinology, and Metabolism, Churchill Hospital, Oxford OX3 7lJ, UK; Email: leanne.hodson@oxlip.ox.ac.uk

**Keywords:** serum fatty acids, nutrition surveys, reference ranges, biomarkers

## Abstract

Serum fatty acids are increasingly used in cross-sectional surveys and cohort studies as biomarkers of dietary fat intake; however, it is currently difficult to judge whether an individual has low or high fatty acid status, or whether the distribution of fatty acids of a group of people is low or high due to a lack of appropriate reference values. In the absence of interpretive criteria, the distribution of serum fatty acids from a suitable reference population can be used as an alternative. We describe the distribution of the fatty acid composition of the three most commonly reported lipid classes in serum; cholesterol ester, phospholipid and triacylgycerol. Results for each serum lipid class are presented as means (SD) and percentiles (5, 10, 25, 50, 75, 90, and 95) of serum fatty acids in non-fasting blood samples collected from a population based cross-sectional survey of New Zealand adults (*n* = 2793). These serum fatty acid reference ranges are applicable and relevant to Australia, United Kingdom, and United States as well as other countries where fat intakes are similar to New Zealand.

## 1. Introduction

The nutritional status of populations is normally assessed using a range of dietary, anthropometric and biochemical measurements. Interpretive criteria for these measures are used to judge the adequacy of nutritional status. Cutpoints or reference values which define the stages of deficiency, insufficiency, adequacy, or excess are the most common interpretive criteria. However, when scientific evidence is insufficient to establish cutpoints for a particular measurement, the distribution range of the measurement in the population can be used to judge if status is high or low for individuals or groups.

A number of serum fatty acids are valid biomarkers of dietary fat intake, and their measurement is becoming a complementary tool in nutrition surveys to monitor the types of fats consumed and in prospective studies designed to generate knowledge about the relation between various types of dietary fat and disease risk [1]. Unfortunately, there is little in the way of interpretive criteria or distribution reference ranges to judge serum fatty acid status of the individual. In general, serum fatty acid composition, given as the molecular percentage of total fatty acids (mol%), or as a weight percent, tends to be reported as mean and standard deviation. We have previously reviewed and summarised this literature [1]; however, virtually all of the results came from studies in which the participants were not randomly selected from the population, thus, there is a need for data which can be generalised to national populations.

The usefulness of representative population-based serum fatty acid data reported as percentiles is obvious because it will enable researchers and epidemiologists to evaluate and judge what constitutes high or low fatty acid status. For example, what constitutes a high or low value for serum linoleic acid, the predominant polyunsaturated serum fatty acid, or serum myristic acid, a marker of dairy fat intake in a reference population?

In this paper we report the percentiles of serum phospholipid, cholesterol ester, and triacylglycerol fatty acids from a nation-wide representative survey of New Zealand adults.

## 2. Methods

### 2.1. Survey Methods

All participants in the New Zealand 1997 National Nutrition survey (NNS97) were recruited from the 1996/1997 New Zealand Health Study (NZHS). Ethical approval for the surveys was obtained from the 14 ethics committees throughout New Zealand, and all participants (or the guardians of those less than 18 years old) gave informed written consent. The methodology for both surveys has been described in detail elsewhere [2,3,4]. Briefly, a three stage stratified design was used in the 1996/1997 NZHS. New Zealand was divided into 18,880 small geographical areas. These primary sampling units (PSU) were divided into 122 strata, based on various geographical and socioeconomic characteristics. The first stage of the survey involved selection of PSUs. Secondly, households within the PSUs were randomly chosen. Finally, one eligible adult from the chosen household was randomly selected to participate in the NZHS. All participants who consented to participate in the 1996/1997 NZHS were invited to participate in the NNS97 which was to follow several months later than the NZHS. Those who consented to participate in NNS97 were interviewed in their homes. During the home-interview, height and weight were measured, and body mass index (BMI, kg/m^2^) calculated. Smoking status and ethnicity were self-reported. Also during this interview, NNS97 participants completed a computer‑assisted 24 h dietary recall, and were asked to provide a blood sample. The NNS97 participants were not asked to fast before the blood sample was collected. Blood was taken into a 10 mL vacuum evacuated tube with no anticoagulant and tubes immediately placed in a polystyrene container which contained an ice pack. The container was sent on the same day to a laboratory for processing and storage of serum. Serum was stored at −80 °C until the fatty acid analysis.

### 2.2. Serum Fatty Acid Analysis

Serum lipids were extracted according to the method of Bligh and Dyer [5]. To separate out lipid classes, the lipid extracts were spotted onto silica gel coated thin layer chromatography plates and placed in a glass tank containing a solvent system of hexane:diethyl ether:acetic acid, in the ratio 85:15:1 by volume. The lipid bands were visualised by spraying the glass plates with 0.1% solution (weight:volume) of 8-anilino-1-napthalene sulfonic acid and viewing them under ultraviolet light. Serum cholesterol ester, phospholipid and triacylglycerol bands were scraped into glass test tubes and the fatty acids were converted into fatty acid methyl esters using 6% sulphuric acid in methanol and incubating at 80 °C for 2 h, for cholesterol ester and triacylglycerol, and 12–16 h, for phospholipid. The samples were then eluted into hexane and stored at −20 °C.

An HP6890 Series Gas Chromatograph (Aligent, Palo Alto, CA) with a DB-225 megabore column (internal diameter: 30 m × 0.25 mm; film thickness: 0.25 µm; J & W Scientific, Deerfield, IL) and flame ionization detection was used to separate fatty acids. Blank extractions were analysed to correct for background contamination. Throughout the analysis, precision was monitored by analysing one pooled serum sample for every 20 NNS97 samples. The coefficients of variation (CVs) for the fatty acid analysis were below 15% for the vast majority of fatty acids in serum cholesterol ester, phospholipid and triacylglycerol (Table 1). The fatty acids are reported as molecular percents (mol%).

### 2.3. Statistial Analysis

STATA (version 9.0; Stata Corp, College Station, TX) was used for all statistical analyses. Differences in the distribution of variables between the NNS97 participants who had a fatty acid measurement of at least one of serum cholesterol ester, phospholipid, or triacylglycerol, and NNS97 participants who did not have a fatty acid measurement, were tested using *t*-tests for continuous variables and chi-squared tests for categorical variables. The major fatty acids—for which greater than 75% of the study population had a detectable value—in serum cholesterol ester, phospholipid and triacylglycerol are presented. For the serum fatty acid analyses, survey commands were used to take into account the complex survey design of the NNS97. The survey-adjusted mean (SD) as well as percentiles were calculated for each of the major fatty acids.

**Table 1:** Coefficients of Variation (CVs) for the serum fatty acid analysis.

			CV		
		Fatty acid	Cholesterol ester	Phospholipid	Triacylgycerol
SAFA		14:0	15.0%	22.7%	15.1%
		15:0	12.0%	11.2%	15.0%
		16:0	7.8%	7.5%	4.4%
		18:0	15.4%	7.9%	12.8%
		20:0	-	14.9%	-
		22:0	-	12.8%	-
		24:0	-	15.4%	-
					
MUFA		16:1n*-*7	16.5%	23.2%	11.2%
		18:1n*-*9	11.4%	4.4%	6.1%
		20:1n*-*9	-	-	32.6%
		24:1n*-*9	-	10.6%	-
					
PUFA	n*-*3	18:3n*-*3	14.3%	10.6%	25.6%
		20:5n*-*3	16.5%	13.9%	18.9%
		22:5n*-*3	-	12.0%	-
		22:6n*-*3	25.4%	13.1%	22.5%
				
	n*-*6	18:2n*-*6	3.0%	10.6%	4.0%
		18:3n*-*6	9.6%	-	17.3%
		20:3n*-*6	4.6%	9.5%	35.7%
		20:4n*-*6	4.9%	10.7%	21.6%
		22:4n*-*6	-	-	32.2%

SAFA: saturated; MUFA: monounsaturated; PUFA: polyunsaturated.

## 3. Results and Discussion

In total, 7862 people—a response rate of 73.8%—participated in the NZHS. Of these participants, 4636 completed a 24 h recall for the NNS97, and 69.5% of the NNS97 participants (*n* = 3228) consented to blood samples. The fatty acid composition of serum cholesterol ester, phospholipid and triacylglycerol was analysed for 2393, 2416, and 2402 participants, respectively. The fatty acid composition of at least one of the serum lipid classes was obtained from 2793 participants.

Participant characteristics are presented in Table 2. It has previously been reported that the 1996/1997 sample (*n* = 7862) was similar to the NNS97 sample (*n* = 4636) with respect to the distribution of sex, age, ethnicity, marital status, labourforce status and smoking status [3]. The *P* values presented in Table 2 are from tests comparing the characteristics of the NNS97 participants who had the fatty acid composition of at least one of serum cholesterol ester, phospholipid, or triacylglycerol measured, and the NNS97 participants that did not have any serum fatty acid measurement. There were small but statistically significant differences in the distribution of sex, age, ethnicity, and BMI between NNS97 participants who had a fatty acid measurement and NNS97 participants who did not. There were no significant differences in smoking status, serum cholesterol concentrations, or in the intakes of dietary fat between these two groups. Most of the participants were between the ages of 25 to 44 years. Approximately one third of the participants were overweight, with an additional fifth classified as obese. Just over a quarter of the participants were current smokers. Sixteen percent of participants reported consuming fish in the previous 24 h. Dairy fat accounted for 20% of the total dietary fat (data not shown). Fat intakes and major food sources of fat did not differ between participants who had a fatty acid value and participants who did not (Table 2). 

**Table 2:** Characteristics of the participants of the 1996/1997 NZHS and the 1997 National Nutrition Survey.

			NNS97 participants			
	1996/1997 NZHS	NNS97 participants	Fatty acid measurement ^1^	No fatty acid measurement		*P*^2^
*n* (%) of 1996/1997 NZHS	7862 (100.0)	4636 (59.0)	2793 (35.5)	1843 (23.4)		
						
Females	4604 (58.6)	2709 (58.4)	1547 (55.4)	1162 (63.0)		<0.001
						
Age category (y)						
15 to 18	405 (5.2)	246 (5.3)	122 (4.4)	124 (6.7)		0.001
19 to 24	645 (8.2)	354 (7.6)	210 (7.5)	144 (7.8)		
25 to 44	3221 (41.0)	1964 (42.4)	1227 (43.9)	737 (40.0)		
45 to 64	2063 (26.2)	1255 (27.1)	765 (27.4)	490 (26.6)		
65+	1528 (19.4)	817 (17.6)	469 (16.8)	348 (18.9)		
						
Ethnicity						
NZEO	5896 (75.0)	3626 (78.2)	2286 (81.8)	1340 (72.7)		<0.001
NZ Maori	1321 (16.8)	703 (15.2)	368 (13.2)	335 (18.2)		
Pacific people	646 (8.2)	307 (6.6)	139 (5.0)	168 (9.1)		
						
BMI category						
Normal	-	1882 (40.6)	1189 (42.6)	693 (37.6)		<0.001
Overweight	-	1530 (33.0)	999 (35.8)	531 (28.8)		
Obese	-	967 (20.9)	551 (19.7)	416 (22.6)		
Not measured	-	257 (5.5)	54 (1.9)	203 (11.0)		
						
Current smokers	2280 (29.0)	1258 (27.1)	763 (27.3)	495 (26.9)		0.730
						
Total cholesterol (mmol/L) ^3^	-	5.8 (1.2)	5.8 (1.2)	5.8 (1.2)		0.6876
						
HDL cholesterol (mmol/L) ^3^	-	1.3 (0.4)	1.3 (0.4)	1.3 (0.4)		0.5642
						
Dietary fat intake (% of kJ) ^3^						
Total fat	-	34.27 (11.90)	34.41 (8.74)	34.32 (9.12)		0.7416
Saturated fat	-	14.68 (5.10)	14.72 (5.01)	14.61 (5.22)		0.4845
Dairy fat	-	7.38 (6.87)	7.49 (6.88)	7.23 (6.84)		0.2173
Monounsaturated fat	-	11.37 (3.62)	11.38 (3.55)	11.37 (3.71)		0.9575
Polyunsaturated fat	-	4.85 (2.49)	4.83 (2.47)	4.87 (2.52)		0.6573
Fish fat	-	0.62 (2.45)	0.61 (2.33)	0.64 (2.63)		0.6582

NZHS, New Zealand Health Survey; NNS97, New Zealand National Nutrition Survey; CE, cholesterol ester; PL, phospholipid; TAG, triacylglycerol; NZEO, New Zealand European or other; NZ, New Zealand; ^1^ NNS97 participants who had the fatty acid composition of at least one of serum cholesterol ester, phospholipid or triacylglycerol measured; ^2^ For the difference between NNS97 participants who had a fatty acid value (*n* = 2793) and those who did not (*n* = 1843). Differences were tested using chi-squared tests for categorical variables and *t*-tests for continuous variables; Values are number (%) unless otherwise indicated; ^3^ Mean (SD).

Table 3, Table 4 and Table 5 report the fatty acid composition of serum cholesterol ester, phospholipid and triacylglycerol as means (SD) and percentiles. We describe the following fatty acid results: myristic acid (14:0) and pentadecanoic acid (15:0) as they are good biomarkers of dairy and saturated fat intake; linoleic acid (18:2n*-*6) as it is the major polyunsaturated fatty acid in the diet and in serum; and the major long chain n-3 polyunsaturated fatty acids, eicosapentaenoic acid (20:5n*-*3) and docosahexaenoic acid (22:6n*-*3).

The proportion of myristic acid was highest in serum triacylglycerol followed by serum cholesterol ester and phospholipid. The median (10th and 90th percentile) of myristic acid in serum triacylglycerol was 3.36 mol% (1.83 and 5.67 mol%), 1.01 mol% (0.61 and 1.57 mol%) in cholesterol ester, and 0.50 mol% (0.33 and 0.72 mol%) in phospholipid.

Pentadecanoic acid was present in smaller amounts than myristic acid in all three lipid classes. The median (10th and 90th percentile) of pentadecanoic acid was highest in serum triacylglycerol at 0.51 mol% (0.33 and 0.80 mol%) and similar in phospholipid and cholesterol ester at 0.29 (0.20 and 0.39 mol%) and 0.28 mol% (0.19 and 0.40 mol%), respectively.

Linoleic acid was the major fatty acid in serum cholesterol ester, and the major polyunsaturated fatty acid in phospholipid and triacylglycerol. The median contribution (10th and 90th percentile) was 50.30 mol% (41.02 and 57.42 mol%) in cholesterol ester, 19.25 mol% (14.56 and 23.31 mol%) in phospholipid, and 13.38 mol% (8.18 and 21.08 mol%) in triacylglycerol.

The percentiles of eicosapentaenoic acid in serum cholesterol ester and phospholipid were similar with a median (10th and 90th percentile) value of 0.96 mol% (0.50 and 1.66 mol%) in cholesterol ester and 0.92 mol% (0.51 and 1.53 mol%) in phospholipid. The proportion of eicosapentaenoic acid in triacylglycerol was lower; the median contribution (10th and 90th percentile) was 0.23 mol% (0.12 and 0.42 mol%).

The median contribution of docosahexaenoic acid was highest in serum phospholipid at 2.55 mol% and similar in cholesterol ester (0.48 mol%) and triacylglycerol (0.42 mol%). For the central 80% (10th to 90th percentile) of the participants, docosahexaenoic acid contributed between 1.63 and 3.70 mol% for phospholipid, 0.32 and 0.72 mol% for cholesterol ester, and 0.21 and 0.84 mol% for triacylglycerol.

**Table 3:** National Nutrition Survey participant percentiles for serum cholesterol ester fatty acid composition.

			Mean (SD)		Percentile						
		Fatty acid	*n* = 2393		5	10	25	50	75	90	95
SAFA		14:0	1.05 (0.55)		0.51 ^a^	0.61	0.78	1.01	1.28	1.57	1.72
		15:0	0.29 (0.13)		0.17	0.19	0.23	0.28	0.33	0.40	0.46
		16:0	12.12 (2.86)		9.88	10.37	11.07	11.82	12.78	13.91	15.50
		18:0	0.95 (0.42)		0.57	0.63	0.75	0.89	1.09	1.35	1.54
											
MUFA											
		16:1n-7	4.35 (2.33)		2.00	2.41	3.13	4.03	5.26	6.70	7.89
		18:1n-9	19.36 (4.54)		14.58	15.52	17.23	19.18	21.22	23.51	24.83
										
											
PUFA	n*-*6	18:2n-6	49.58 (9.94)		37.08	41.02	46.00	50.30	54.01	57.42	59.82
		18:3n-6	1.04 (0.51)		0.48	0.58	0.76	0.98	1.28	1.59	1.77
		20:3n-6	0.67 (0.20)		0.46	0.49	0.57	0.66	0.76	0.87	0.94
		20:4n-6	5.58 (1.75)		3.59	3.98	4.70	5.49	6.39	7.21	7.84
										
	n-3	18:3n-3	0.76 (0.32)		0.45	0.50	0.60	0.73	0.89	1.05	1.17
		20:5n-3	1.03 (0.60)		0.42	0.50	0.70	0.96	1.28	1.66	1.91
		22:6n-3	0.50 (0.21)		0.28	0.32	0.38	0.48	0.59	0.72	0.80

SAFA, saturated; MUFA, monounsaturated; PUFA, polyunsaturated; Mean (SD) and percentiles are survey adjusted; ^a^ Fatty acids are expressed as a molecular percent (mol%).

**Table 4:** National Nutrition Survey participant percentiles for serum phospholipid fatty acid composition.

			Mean (SD)		Percentile						
		Fatty acid	*n* = 2416		5	10	25	50	75	90	95
SAFA		14:0	0.52 (0.24)		0.30 ^a^	0.33	0.41	0.50	0.60	0.72	0.80
		15:0	0.29 (0.10)		0.18	0.20	0.24	0.29	0.34	0.39	0.41
		16:0	31.73 (4.22)		28.41	28.98	29.96	31.06	32.78	35.02	37.56
		18:0	14.34 (2.59)		11.95	12.49	13.22	14.05	15.06	16.42	17.79
		20:0	0.55 (0.16)		0.38	0.42	0.48	0.54	0.61	0.69	0.75
		22:0	1.54 (0.45)		1.08	1.18	1.32	1.50	1.72	1.95	2.11
		24:0	1.28 (0.42)		0.89	0.96	1.08	1.23	1.42	1.64	1.80
											
MUFA		16:1n-7	0.83 (0.45)		0.38	0.47	0.59	0.78	1.01	1.27	1.43
		18:1n-9	10.00 (2.51)		7.57	8.13	8.90	9.86	11.08	12.11	13.00
		24:1n-9	1.73 (0.54)		1.09	1.26	1.47	1.71	1.99	2.21	2.42
											
PUFA	n-6	18:2n-6	19.01 (5.22)		12.72	14.56	16.95	19.25	21.36	23.31	24.54
		20:3n-6	0.09 (0.08)		1.57	1.83	2.20	2.61	3.07	3.50	3.76
		20:4n-6	7.16 (2.19)		4.44	5.25	6.28	7.18	8.20	9.11	9.70
										
	n-3	18:3n-3	0.24 (0.12)		0.12	0.14	0.18	0.23	0.30	0.37	0.42
		20:5n-3	1.00 (0.60)		0.40	0.51	0.71	0.92	1.22	1.53	1.80
		22:5n-3	0.89 (0.32)		0.47	0.59	0.75	0.91	1.05	1.18	1.28
		22:6n-3	2.61 (1.07)		1.32	1.63	2.05	2.55	3.12	3.70	4.09

SAFA, saturated; MUFA, monounsaturated; PUFA, polyunsaturated; Mean (SD) and percentiles are survey adjusted; ^a^ Fatty acids are expressed as a molecular percent (mol%).

**Table 5:** National Nutrition Survey participant percentiles for serum triacylglycerol fatty acid composition.

			Mean (SD)		Percentile						
		Fatty acid	*n* = 2402		5	10	25	50	75	90	95
SAFA		14:0	3.62 (2.33)		1.43 ^a^	1.83	2.48	3.36	4.48	5.67	6.53
		15:0	0.54 (0.34)		0.29	0.33	0.41	0.51	0.65	0.80	0.87
		16:0	29.22 (5.77)		23.00	24.40	26.63	29.02	31.53	34.01	36.03
		18:0	4.89 (2.40)		2.88	3.25	3.79	4.61	5.65	6.94	7.86
		20:0	0.09 (0.25)		0.00	0.00 ^b^	0.05	0.07	0.10	0.15	0.18
											
MUFA		16:1n-7	4.82 (2.28)		2.43	2.91	3.67	4.67	5.83	6.94	7.72
		18:1n*-*9	34.36 (5.90)		28.03	29.65	32.05	34.43	37.01	39.22	40.84
		20:1n-9	0.24 (0.17)		0.00 ^c^	0.14	0.19	0.24	0.29	0.34	0.38
											
PUFA	n*-*6	18:2n-6	14.15 (7.65)		6.96	8.18	10.32	13.38	17.28	21.08	24.02
		18:3n-6	0.38 (0.32)		0.13	0.18	0.24	0.34	0.46	0.64	0.75
		20:3n-6	0.32 (0.45)		0.12	0.15	0.20	0.26	0.35	0.47	0.61
		20:4n-6	1.03 (0.75)		0.48	0.57	0.72	0.93	1.21	1.61	1.88
		22:4n-6	0.12 (0.28)		0.00 ^d^	0.05	0.08	0.10	0.13	0.16	0.20
										
	n-3	18:3n-3	0.98 (0.95)		0.27	0.43	0.66	0.91	1.19	1.48	1.75
		20:5n-3	0.26 (0.22)		0.10	0.12	0.17	0.23	0.31	0.42	0.52
		22:6n-3	0.50 (0.55)		0.16	0.21	0.29	0.42	0.59	0.84	1.08

SAFA, saturated; MUFA, monounsaturated; PUFA, polyunsaturated; Mean (SD) and percentiles are survey adjusted; ^a^ Fatty acids are expressed as a molecular percent (mol%); ^b^ 9% of participants had values under the detectable limit; ^c^ 5% of participants had values under the detectable limit; ^d^ 6% of participants had values under the detectable limit.

The results of this study describe the distribution of serum fatty acids, measured as percent of total fatty acids (mol%), in a large, randomly selected sample of adult New Zealanders. The percentiles of fatty acids in serum cholesterol ester, phospholipid and triacylglycerol provide population-based reference ranges against which the fatty acid status of individuals or groups can be compared. For example, with percentile reference ranges it is possible to judge where an individual’s eicosapentaenoic acid level ranks relative to a population-based sample. It is our experience that these reference ranges will be useful to researchers and epidemiologists. 

Epidemiological studies which use fatty acids as biomarkers of dietary fat intake or disease risk commonly measure the fatty acid composition of serum or plasma. In this regard, the fatty acid percentiles that we have described for serum are equally applicable to plasma given that Moilanen and Nikkari [6] found no difference in the fatty acid composition of serum or plasma.

The distribution of serum fatty acids values in a population is influenced by a number of factors but clearly, the most important determinant is the range of dietary fat intakes in the population. These reference fatty acid percentiles are therefore relevant to other countries that have similar fat intakes to New Zealanders. This includes—but is not limited to—populations from Australia, the United Kingdom and the United States; the results of recent national nutrition surveys in these countries indicate that in adults the saturated, monounsaturated and polyunsaturated fat intakes respectively contribute 13%, 12% and 5% of total energy in Australia [7], 12%, 12% and 6% of total energy in the United Kingdom [8] and 11%, 13% and 7% of total energy in the United States [9,10].

It is noteworthy that the mean serum fatty acid values from our population based data are similar to the average serum fatty acid composition reported by Hodson *et al.* [1]. The serum fatty acid values summarised by Hodson *et al.* [1] are drawn from studies carried out in a range of different countries, most of which used non-randomly selected convenience samples of participants. This indicates that the range in our population based fatty acid percentiles varies enough so as to be generalizable to a wide range of populations. Although it should be noted that countries which differ markedly from New Zealand in the proportion of saturates and unsaturates in the diet may have slightly different normative distributions for some serum fatty acids. For example, populations with high intakes of fish—such as the Japanese population—would be expected to have a higher proportion of serum n-3 long-chain polyunsaturated fatty acids. 

Non-dietary factors—such as sex [11,12,13,14], age [11,15], smoking [16,17,18,19], adiposity [12], and exercise [20]—can also affect fatty acid composition. In comparison to diet, however, their influence is minor and an individual’s sociodemographic characteristics would not appreciably change the interpretation of their fatty acid status.

One of the main strengths of this study is that the participants were randomly selected from a nationwide sampling frame. There were some small differences in sociodemographic characteristics between the participants who had a fatty acid measurement and the participants who did not, but importantly, there were no differences in dietary fat intakes between these two groups. Thus, the serum fatty acid results are representative of the rest of the survey population. It is worth noting that the NNS97 participants provided non-fasting blood samples and that the precisions of measurement for some of the minor fatty acids were higher than 15%. In the postprandial state, the appearance of chylomicrons in systemic circulation can influence serum fatty acid composition; however, the effect of recent fat ingestion is largely confined to serum triacylglycerol fatty acids and the magnitude of change will depend on the amount of fat in the meal and to what extent the fat differs in composition from usual intake [21,22]. Whilst for an individual there may be discrepancies between the fat content of a recent meal and the usual diet, at a population level it will be similar. Thus, these factors should not have affected the mean fatty acid values but may have broadened the distribution across percentiles. Nevertheless, the results are relevant, particularly given that most large epidemiological studies, such as the European Prospective Investigation into Cancer and Nutrition and the UK Biobank study, also collect non-fasting blood samples for fatty acid analysis [23,24].

The choice of serum lipid class in which to measure fatty acids depends on a large number of factors. For example, is the purpose of the research to assess fat intake or to predict disease risk, to examine saturated fat intake or long chain n-3 polyunsaturated fatty acids? There is no one class of serum lipid that will serve all purposes; however, for particular outcomes some lipid classes may perform better than others. Some of these issues have been addressed elsewhere [1,25].

## 4. Conclusions

This study is the first to present reference ranges for the fatty acid composition of the three most common serum lipid classes: cholesterol ester, phospholipid, and triacylglycerol from a large national survey which used population-based sampling techniques. These serum fatty acid percentile reference ranges will be a useful tool for researchers and epidemiologists in guiding the interpretation of the fatty acid status of adult study populations from a wide range of countries.
